# Dendritic Spine Plasticity: Function and Mechanisms

**DOI:** 10.3389/fnsyn.2020.00036

**Published:** 2020-08-28

**Authors:** Karen Runge, Carlos Cardoso, Antoine de Chevigny

**Affiliations:** Institut de Neurobiologie de la Méditerranée (INMED) INSERM U1249, Aix-Marseille University, Marseille, France

**Keywords:** dendritic spine plasticity, molecular controls, neurodevelopmental disorders, two photon imaging, structural plasticity

## Abstract

Dendritic spines are small protrusions studding neuronal dendrites, first described in 1888 by Ramón y Cajal using his famous Golgi stainings. Around 50 years later the advance of electron microscopy (EM) confirmed Cajal’s intuition that spines constitute the postsynaptic site of most excitatory synapses in the mammalian brain. The finding that spine density decreases between young and adult ages in fixed tissues suggested that spines are dynamic. It is only a decade ago that two-photon microscopy (TPM) has unambiguously proven the dynamic nature of spines, through the repeated imaging of single spines in live animals. Spine dynamics comprise formation, disappearance, and stabilization of spines and are modulated by neuronal activity and developmental age. Here, we review several emerging concepts in the field that start to answer the following key questions: What are the external signals triggering spine dynamics and the molecular mechanisms involved? What is, in return, the role of spine dynamics in circuit-rewiring, learning, and neuropsychiatric disorders?

## Introduction

Dendritic spines are the postsynaptic sites of most excitatory synapses, found along the dendrites of neurons. *Ramón y Cajal in 1888* was the first to observe these small protrusions 1.0–1.5 μm in length in Golgi stainings (Cajal, 1888). He proposed them to be points of contact between neurons. Towards the end of the century, theories emerged proposing that changes in brain activity and function could be driven by morphological modifications of spines (reviewed by DeFelipe, 2015). Following years of speculations, it was only in 1959 with the development of electron microscopy (EM) that spines were confirmed to be the points of contact between neurons, by forming the postsynaptic element of synapses (Gray, 1959). Comparing brain tissue at immature vs. old ages (Feldman and Dowd, 1975) or after being exposed to an enriched or impoverished environment (Globus et al., 1973) showed striking differences in spine densities, indicating that dendritic spines must be to some degree plastic. Ziv and Smith (1996) and Fischer et al. (1998) eventually observed dendritic spine dynamics for the first time in cultured hippocampal neurons and were intrigued by the unexpected rapidity of spine formation and elimination. With the development of two-photon microscopy (TPM; Denk et al., 1990) and the emergence of transgenic animals expressing fluorochromes in neurons in the early 2000s (Feng et al., 2000; Keller-Peck et al., 2001), researchers became able to follow spine changes over time *in vivo* (Grutzendler et al., 2002; Trachtenberg et al., 2002). These revolutionizing studies gathered information on spine dynamics in basal vs. specific contexts, for example, motor tasks (Xu et al., 2009) or sensory deprivations (Zuo et al., 2005a; Keck et al., 2008). Nowadays, *in vivo* TPM has gained in precision owing to improvements in optical tools and the ability to express high-quality fluorophores in defined subsets of neurons. In addition to the use of transgenic mouse lines, e.g., Thy1-GFP (Feng et al., 2000), other techniques as viral transmission (Kuhlman and Huang, 2008), *in utero* electroporation (Villa et al., 2016; Subramanian et al., 2019) and single-cell electroporation (Pagès et al., 2015) have allowed controlled spatial and temporal expression of fluorescent dyes and other genetic constructs in desired cell types across the cortex.

To excite the target neurons with a two-photon laser within the living brain, one has to remove or thin a part of the animal’s skull. Since the beginnings of *in vivo* TPM, two techniques have emerged. The first is cranial window surgery (Trachtenberg et al., 2002; Villa et al., 2016), where a piece of bone is removed and replaced with a transparent window. The second is thinned-skull cranial window surgery (Grutzendler et al., 2002; Isshiki et al., 2014), where, in an attempt to be less invasive, the skull is thinned using micro-surgical blades to a thickness of approximately 20 μm (Zuo et al., 2005b), rendering the bone translucent. While cranial window implantation has been associated with inflammation-induced spine turnover (Xu et al., 2007) for more than 20 days post-surgery, cranial thinning in turn does not require a recovery period, is less associated with inflammation and allows immediate imaging (Yang et al., 2010). However, the thinned skull technique is more challenging and, due to the natural regrowth of the thinned bone, one has to re-thin the window to image repeatedly (Zuo et al., 2005b). Nowadays, both techniques are largely employed. For more details on the cranial window and thinned-skull cranial window surgeries please refer to Xu et al. (2007) and Yang et al. (2010).

In the last decades, spine dynamics have taken a prominent role in explaining the brain’s adaptability. Deciphering the intrinsic and extrinsic mechanisms that underlie specific turnover properties shall help to elucidate the functional changes that in turn lead to complex cognitive abilities or drive pathological outcomes. Here, we review the most recent literature in the field of spine dynamics with a particular focus on *in vivo* TPM. We address the following questions: What are the intrinsic and experience-dependent signals that control spine dynamic and how? What is, in return, the role of spine-dynamics in behavior?

## Spinogenesis and Spine Subtypes

For a list of the main studies with *in vivo* TPM of spine dynamics during development, see Table 1. Spines are commonly classified into filopodia, stubby, thin, and mushroom spines, according to their shape and size (Peters and Kaiserman-Abramof, 1970). These fixed categories have been challenged, though, as in physiological conditions spines are constantly evolving and morphological stages are transitory (Tønnesen et al., 2014).

**Table 1:** Spine plasticity during development.

Brain region	Age animals	Impact on spine stability	Main results	Methods	Reference
**Visual cortex**					
Visual cortex, PN, Layer 5	P30	73% stable spines over 30 days	Spines become more stable from juvenile to adult ages.	Two-photon laser scanning, Thinned skull cranial window, YFP-labeled dendrites	Grutzendler et al. (2002)
	4 months	96% stable spines over 30 days		
Visual cortex, auditory cortex, somatosensory cortex, PN, Layer 5	P40-P61	VC: 78% of spines stable over 3 weeks SS: 88% of spines stable over 3 weeks AC: 89% of spines stable over 3 weeks	Most (~80%) spines in the cortex are stable over 3 weeks.	Two-photon laser scanning, Thinned skull cranial window, GFP or YFP-labeled dendrites	Majewska et al. (2006)
**Somatosensory cortex**				
Somatosensory cortex, visual cortex, PN, Layer 5	P16-25	SS: 35% stable spines over ≥8 days	Spines become more stable from juvenile to adult ages. Stability in SS and VC is similar.	Two-photon laser scanning, Craniotomy, GFP or YFP-labeled dendrites	Holtmaat et al. (2005)
	P35-80	SS: 54% stable spines over ≥8 days		
	P80-120	SS: 66% stable spines over ≥8 days		
	P175-225	SS: 73% stable spines over ≥8 days		
	3-6 months	VC: 75% stable spines over ≥8 days		
Barrel cortex, Motor cortex, Frontal cortex, PN Layer 5	P30	60% stable over 22 months	Spines become more stable in adulthood and a majority of them can last throughout life.	Two-photon laser scanning, Thinned skull cranial window, YFP-labeled dendrites	Zuo et al. (2005b)
	4-6 months	74% stable over 18 months		
Barrel cortex, PN, Layer 2/3	P56	78% of stable spines observed over 17 days contain a PSD	Most stable spines have a PSD.	Two-photon laser scanning, Craniotomy, DsRedExpress-labeled dendrites, GFP-labeled PSD-95	Cane et al. (2014)

Filopodia are long and thin protrusions without bulbous heads whose contribution is high in early postnatal life and rapidly drops down in adulthood. They are the most dynamic dendritic protrusions as they can appear and disappear in as little as 10 min (Ziv and Smith, 1996). Thin spines have small heads separated from the dendrite by long, thin necks. Stubby spines were initially described as containing a bulbous head directly budding from the dendrite without intermediate neck structures. However, recent superresolution imaging based on stimulated emission depletion (STED) microscopy has indicated that stubby spines in fact would have very short necks connecting the head to the dendrite and that those short necks would be visible only by STED (Tønnesen et al., 2014). Further time-lapse experiments suggested that mushroom spines undergo neck length reduction upon stimulation, indicating that stubby spines could be a form of active mushroom spines with very short necks (Tønnesen et al., 2014). Thus, the structural and functional roles of stubby spines need to be reevaluated. Stubby and thin spines are less dynamic than filopodia and can persist over several days (Holtmaat et al., 2005). The least dynamic spines are the large-headed mushroom spines that can be stable over several months (Grutzendler et al., 2002). Mushroom spines are commonly seen as functional spines and synaptically connected to an axonal bouton. Following stimulation, thin spines were shown to acquire a full functional synapse and transit simultaneously into stable mushroom spines (Matsuzaki et al., 2004); this need to be reevaluated now that some stubby spines are mushroom spines with short necks (Tønnesen et al., 2014). However, the presence of synapses in small spines does not always predict stability nor the future acquisition of a mushroom morphology. Using *in vivo* TPM imaging and EM, it has been shown that fractions of small transient spines are also able to form temporary synapse components and to participate in functional circuits before being eliminated (Cane et al., 2014).

Whether spine formation reflects some intrinsic properties of postsynaptic dendrites that precede synaptogenesis or if it is induced by extrinsic factors associated with presynaptic axonal terminals during synaptogenesis is still under debate. However, strong lines of evidence indicate that dendritic filopodia are involved in the initial stages of spinogenesis and synaptogenesis in most, if not 100%, of cases. First, time-lapse observations from neuron cultures and brain organotypic slices have revealed that dendritic filopodia can initiate contacts with presynaptic axons and are occasionally transformed into spines (Dailey and Smith, 1996; Ziv and Smith, 1996). Second, the presence of synaptic contacts between a fraction of filopodia and axons was confirmed by EM studies (Fiala et al., 1998). These findings suggest that filopodia are spine precursors acting as samplers of the local synaptic neighborhood. Later, *in vivo* imaging in YFP-expressing young mice demonstrated that dendritic filopodia are indeed highly dynamic and can transform into spines (Grutzendler et al., 2002; Zuo et al., 2005b). In juvenile mice, ~12% of all dendritic protrusions in different cortical regions are filopodia, the remaining being spines. Whereas most filopodia at a given time point underwent rapid turnover within a few hours, ~15% rapidly transformed into spine-like protrusions, of which 20% survived long term (Zuo et al., 2005b). These newly persistent protrusions were morphologically indistinguishable from preexisting spines. In sum, in the brain of young mice, a small percentage of filopodia observed at a given time point are transformed into stable thin or mushroom-like dendritic spines, while other filopodia are eliminated. These *in vivo* observations reinforce the notion that dendritic filopodia are spine precursors that sample the environment in search of axonal partners to elicit spinogenesis and synaptogenesis.

Although the aforementioned data indicate that spines are born from filopodia that have found a presynaptic axonal partner during dynamic sampling, several studies have suggested that other modes of spinogenesis might also occur at specific developmental time points. First, EM studies in early development have suggested that excitatory shaft synapses precede the formation of spine synapses (Fiala et al., 1998; Zuo et al., 2005b). These observations were reinforced by time-lapse imaging studies showing that at this stage spines can form directly from dendritic shafts without passing by a filopodial stage (Dailey and Smith, 1996). Such observations were confirmed more recently in studies showing that glutamate and γ-aminobutyric acid (GABA) uncaging can induce spine formation without the need for a filopodial intermediate (Kwon and Sabatini, 2011; Oh et al., 2016). These observations suggest that spinogenesis during very early development might skip a filopodial sampling phase, although the requirement for presynaptic axon proximity seems preserved.

Overall, the current knowledge strongly supports the hypothesis that, at least in young mice, axonal growth and neurotransmitter release may be the triggering events in dendritic spine formation such that axonal bouton localization and activity are important triggers of spinogenesis.

## Spine Pruning in Youth, Stability in Adulthood

Since the first studies on fixed tissues, striking differences in spine densities were observed between developmental ages. Across several mammalian species, including humans, projection neurons in young brains show a much higher spine density compared to those in adults (Rakic et al., 1986; Markus and Petit, 1987; Lübke and Albus, 1989; Huttenlocher, 1990; Duan et al., 2003). This suggests that during youth, neuronal networks undergo significant modifications mediated, at least in part, by synapse elimination. However, these analyses on fixed postmortem tissues did not allow determining the dynamic behavior of dendritic spines. This is important because, as spines are dynamically formed and eliminated over time, the decrease in net spine density with increasing age may be due to an increase in the elimination of existing spines, the addition of fewer new spines, or a combination of both. To address this issue, dendritic spines of layer 5 (L5) neurons were longitudinally imaged by *in vivo* TPM. Such studies showed that in juvenile mice, 13–20% of spines were eliminated and 5–8% were formed over a 2-week interval in barrel, motor, visual and frontal cortical areas, indicating a cortex wide elimination of spines during this developmental period (Grutzendler et al., 2002; Zuo et al., 2005b). Because the amount of spine elimination is more than two-fold higher than that of spine formation between adolescence and adulthood, these *in vivo* studies indicate that the net spine loss observed in fixed tissue is mainly due to the elimination, also called “pruning,” of existing synaptic connections.

The question of the stability of spines in adulthood was first treated in 2002 by two groups who initially came to different conclusions: Trachtenberg et al. (2002) found that in 6–10 week old young adult mice only 60% of spines were stable over 8 days, suggesting a relative instability of adult spines (Trachtenberg et al., 2002). Grutzendler et al. (2002), in contrast, when imaging 4 months old animals, observed a massive 96% of spines to be stable over at least 30 days (Grutzendler et al., 2002). Further quantitative predictions resulted in a spine half-life of more than 13 months, which implies that most adult spines would be stable lifelong. Besides the differences in mouse age and cortical area examined, the differences in spine stability between the two studies likely arose primarily from the use of an open-skull glass window vs. a thinned-skull window for imaging. As mentioned earlier, cranial surgery for an open-skull glass window is associated with strong inflammatory responses that can last up to 20 days and strongly increase spine plasticity (Xu et al., 2007). Trachtenberg et al. (2002) started imaging as early as 7 days post-surgery, which raises the possibility that the observed spine instability was in large part artifactual, due to inflammatory-associated processes. Later TPM studies confirmed the long-term stability of most adult spines (Holtmaat et al., 2005; Zuo et al., 2005b). Overall, although open-skull imaging studies tend to artificially enhance basal spine turnover rates, the consensus is that in adulthood dendritic spines are largely long-term stable.

The above imaging studies were performed on L5 neurons of visual and motor areas. Since, then, other studies have shown that, although globally held, baseline spine dynamics in adulthood can differ to some degree between cortical areas and cell types. At the areal level, basal spine dynamics are reduced in the visual cortex compared to the somatosensory and auditory areas (Majewska et al., 2006). At the laminar level, Tjia et al. (2017) showed that baseline spine turnover is much higher in cortical L2/3 than in L5 and that L5 but not L2/3 neurons undergo spine pruning between juvenile and adult stages (Tjia et al., 2017). Also, learning-induced spine formation is branch-specific in L5 neurons, but this rule does not apply to L2/3 neurons (Ma et al., 2015). Even at the single-cell level, spine dynamics might vary between dendritic compartments. Such variations were reported for example between basal (Gu et al., 2014) and apical dendrites (Attardo et al., 2015) in CA1 pyramidal neurons of the hippocampus, albeit this has to be treated with care as Pfeiffer et al. (2018) demonstrated that insufficient resolution in TPM compared to super-resolution STED microscopy can lead to significant underestimations of spine turnover.

Using *in vivo* TPM, imaging basal spine dynamics of deep-layer neurons have been unachievable due to the high distance from the brain surface. For this reason, most studies on spine dynamics have focused on apical dendrites in L1. The recent advent of three-photon microscopy, however (Ouzounov et al., 2017) shall soon pave the way towards *in vivo* imaging of basal dendrites in cortical neurons.

**Figure 1:**
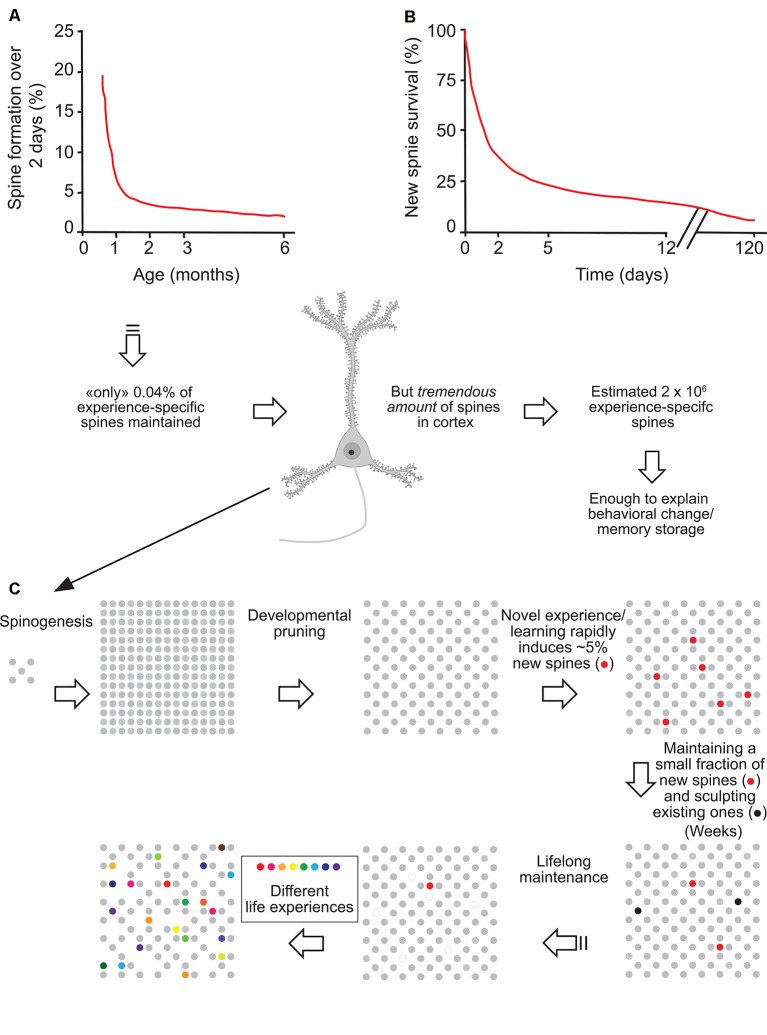
Experience-specific spines constitute a tiny fraction of total spines but can encode memory. **(A)** Spine formation rate declined rapidly from P19 to P30 and remained low thereafter. **(B)** Regardless of animals’ ages, ~5% of new spines formed over 2 days were maintained over a protracted process. **(C)** Schematic summary of spine remodeling and maintenance throughout life. Spines are rapidly formed after birth, undergo experience-dependent pruning during postnatal development, and remain largely stable in adulthood. Learning or novel sensory experience induces the rapid formation of new spines (5% of total spines) within 1–2 days. Only a tiny fraction of new spines (0.04% of total spines) survive the first few weeks in synaptic circuits and are stably maintained later in life. Novel experience also results in the pruning of a small fraction of existing spines formed early during development. New stable spines induced by novel experience, together with existing spines formed during early development and surviving experience-dependent pruning, provide an integrated and stable structural basis for lifelong memory storage, despite ongoing plasticity in synaptic networks. Modified with permission from Yang et al. (2009).

Importantly, although in adulthood most adult spines remain stable and might provide a physical substrate for long-term information storage, the observed small degree of spine turnover, together with rapid changes in synaptic strength of existing spines (Baltaci et al., 2019), may underlie learning and plasticity in the mature brain. Based on formation rates and long-term survival of new spines formed over 2 days, Yang et al. (2009) estimated that the number of such task-specific spines persisting at the end of life should be ~0.04% of the total spines in motor or barrel cortex. However, given the immense quantity of spines in the mouse cortex (~10,000 per neuron), the number of learning-induced and subsequently maintained new spines could be around 2 × 10^6^, large enough to have a significant and lifelong impact on neural network functions and animal’s behavior (Arenz et al., 2008; Houweling and Brecht, 2008; Figure 1).

Although the adult brain is less plastic than the young brain, it is still retaining the fundamental capability of removing spines and forming new ones, which might be essential for the encoding and processing of novel experiences and learning. But how do experiences influence spine dynamics?

## Experience-Dependent Spine Dynamics

Experience-dependent spine remodeling has been shown in a variety of learning tasks and deprivation assays, and the corresponding changes in spine dynamics allowed us to better understand the possible function of spine remodeling in adaptive behaviors, learning, and memory. For a list of the main studies with *in vivo* TPM of spine dynamics during development, see Table 2.

**Table 2:** Experience-dependent spine plasticity.

Modulation	Brain region	Age animals	Impact on spine formation/elimination	Main results	Methods	Reference
**Whisker trimming**					
Chessboard whisker trimming	Barrel cortex, PN, Layer 5	6-10 weeks	Control: 40% stable spines over 4 days Trimmed: 30% stable spines over 4 days	Sensory deprivation increases spine turnover and reduces stability.	Two-photon laser scanning, Craniotomy, GFP-labeled dendrites	Trachtenberg et al. (2002)
Unilateral whisker trimming	Barrel cortex, PN, Layer 5	P30	Control: 17% of spines eliminated and 6% formed over 2 weeks Trimmed: 10% of spines eliminated, 5% formed after 2 weeks	Long-term sensory deprivation in young mice reduces the rate of spine elimination but has no significant effect on spine formation. Spines in adulthood are less affected.	Two-photon laser scanning, Thinned skull cranial window, YFP-labeled dendrites	Zuo et al. (2005a)
		>4 months	Control: 5% of spines eliminated and 4% formed over 2 weeks Trimmed: No changes in spine turnover after 2 weeks			
Chessboard whisker trimming	Barrel cortex, PN, Layer 5	2-5 months	Control: ~63% of spines stable over 28 days Trimmed: ~60% of spines stable over 28 days, turnover increased	Sensory deprivation induces loss of old persistent spines and forms new persistent spines.	Two-photon laser scanning, Craniotomy, GFP-labeled dendrites, Electron microscopy	Holtmaat et al. (2006)
**Motor learning**					
Motor task, Neonatal bilateral whisker trimming	Barrel cortex, Motor cortex, PN, Layer 2/3, Layer 5	P30	Control, MC, L2/3: ~18% spine elimination, ~18% spine formation over 4 days Motor task, MC, L2/3: ~16% spine elimination, ~17% spine formation	Motor task-induced increase in spine dynamics happens only in L5, but not in L2/3 of MC. Neonatal whisker trimming reduces spine formation in L2/3, but not in L5 of the somatosensory cortex.	Two-photon laser scanning, Thinned skull cranial window, GFP-labeled dendrites	Tjia et al. (2017)
			Control, MC, L5: ~9% spine elimination, ~6% spine formation over 4 days Motor task, MC, L5: ~14% spine elimination ~14% spine formation			
			Control, BC, L2/3: ~15% spine elimination, ~15% spine formation After neo. trimming, BC, L2/3: ~17% spine elimination, ~7% spine formation			
			Control, BC, L5: ~12% spine elimination, ~7% spine formation After neo. trimming, BC, L5: ~12% spine elimination, ~7% spine formation			
Forelimb reaching	Motor cortex, PN, L5	P30	Control: ~7% spine elimination, ~5% spine formation over 2 days	Motor learning selectively stabilizes learning-induced new spines into adulthood.	Two-photon laser scanning, Thinned skull cranial window and craniotomy, YFP-labeled dendrites	Xu et al. (2009)
			Reaching: Spine elimination increased after 2 days (~15%), spine formation increased to 11% within 1 h after training			
Rotarod motor task	Motor cortex, PN, Layer 5	P30	Control MC: ~9% elimination, ~7% spine formation over 2 days Rotarod MC: ~9% elimination, ~15% spine formation over 2 days	Learning induces formation of new spines.	Two-photon laser scanning, Thinned skull cranial window, YFP-labeled dendrites	Yang et al. (2009)
		>4 months	Control MC: ~3% elimination, ~3% spine formation over 2 days Rotarod MC: ~4% elimination, ~8% spine formation over 2 days			
**Visual deprivation**						
Monocular deprivation	Visual cortex, PN, Layer 2/3, Layer 5	P45-100	Control L2/3: 8% spine elimination, 7% spines formation over 8 days Control L5: 7% spine elimination, 6% spines formation over 4 days	Visual deprivation increases spine formation.	Two-photon laser scanning, Craniotomy, GFP- labeled dendrites	Hofer et al. (2009)
			MD L2/3: no changes in spine turnover over 4+ days MD L5: spine elimination unchanged, ~11% of spines formed over 4 days			
Monocular- and Binocular deprivation	Visual cortex, PN, Layer 5	P28	Control: ~11% spine elimination, ~8% spine formation over 3 days MD: ~19% spine elimination, ~9% spine formation over 3 days BD: ~10% spine elimination, ~7% spine formation over 3 days	MD over 3 days significantly increases spine elimination without affecting spine formation. BD does not change spine dynamics.	Two-photon laser scanning, Thinned skull cranial window, YFP-labeled dendrites	Zhou et al. (2017)
**Fear conditioning**						
Fear conditioning, Fear extinction	Frontal association cortex, PN, Layer 5	P30	Control: ~18% spine elimination, ~14% spine formation over 9 days Fear cond.: ~23% spine elimination, ~11% spine formation over 9 days Fear ext.: ~10% spine elimination, ~17% spine formation after 2 days	Fear conditioning promotes spine elimination. Fear extinction induces spine formation.	Two-photon laser scanning, Thinned skull cranial window, YFP-labeled dendrites	Lai et al. (2012)
Fear conditioning	Auditory cortex, PN, Layer 5	3-6 months	Control: ~7% spine elimination, ~8% spine formation over 2 h Fear cond.: ~11% spine elimination, ~17% spine formation over 2 h	Auditory fear conditioning causes an increase of spine turnover	Two-photon laser scanning, Craniotomy, GFP-labeled dendrites	Lai et al. (2018)
Fear conditioning	Auditory cortex, PN, Layer 5	7–10 weeks	Control: ~13% spine elimination, ~7% spine formation over 3 days Fear cond.: ~13% spine elimination, ~15% spine formation	Fear conditioning increases formation of new Amygdala–Auditory cortex connections consistent with the consolidation of fear memory.	Two-photon laser scanning, Craniotomy, YFP, tdTomato and GFP-labeled dendrites and axons	Yang et al. (2016)
Fear conditioning, Fear extinction	Auditory cortex, PN, Layer 5	P30	Control: ~9% spine elimination, ~9% spine formation over 3 days Fear cond.: ~10% spine elimination, ~16% spine formation over 3 days Fear ext.: ~17% spine elimination, ~5% spine formation over 2 days	Persistent new spines are induced by auditory fear conditioning. Fear extinction selectively eliminates new spines.	Two-photon laser scanning, Thinned skull cranial window, YFP-labeled dendrites	Lai et al. (2018)
**Stress**					
Corticosterone administration (stress)	Barrel cortex, PN, Layer 5	P23-30	Control: ~4% elimination, ~5% spine formation over 1 day Acute cort.: ~12% elimination, ~7% spine formation over 1 day Chronic cort.: Elimination increases to 22%, Spine formation unchanged over 10 days	Acute corticosterone increases spine turnover. Chronic stress increases spine elimination.	Two-photon laser scanning, Thinned skull cranial window, YFP-labeled dendrites	Liston and Gan (2011)
Motor task, corticosterone administration (stress)	Motor cortex, PN, Layer 5	P30	Untrained: ~7% spine formation over 2 days Training with additional cort: ~17% spine formation over 2 days Chronic cort.: elimination of training associated and pre-training spines over 10 days	Corticosterone increases formation of lasting task-associated spines. Chronic corticosterone causes loss of spines and reduces motor performance.	Two-photon laser scanning, Thinned skull cranial window, YFP-labeled dendrites	Liston et al. (2013)

### Whisker Deprivation in the Barrel Cortex

A group of studies has investigated spine remodeling in the somatosensory barrel cortex, in normal and deprived or stimulated conditions. Naturally, in the juvenile barrel cortex, spine elimination overcomes spine formation, which corresponds to the synaptic pruning that shapes neuronal circuits (Zuo et al., 2005a). Interestingly, sensory deprivation by complete contralateral whisker trimming over 2 weeks in juveniles attenuates spine elimination rates without affecting spine formation, hence reducing the pruning and increasing net spine density (Zuo et al., 2005a; Ma et al., 2015). This indicates that sensory experience is required for synaptic pruning during adolescence. Reproducing complete whisker trimming in adults has a similar, albeit much smaller effect that requires prolonged deprivation (8 weeks of deprivation are required; Zuo et al., 2005a). These studies suggest that experience plays an important role in the net loss of synapses over most of an animal’s lifespan, particularly during adolescence.

In contrast to complete whisker trimming, alternated “chessboard” trimming in juvenile mice increases spine formation over 2 days in the contralateral barrel cortex, while causing no change in elimination rates (Pan et al., 2010). In young adult mice imaged over 1 month, chessboard trimming does not alter net spine density but increases spine turnover by eliminating previously stable spines and stabilizing newly formed ones (Holtmaat et al., 2006). Moreover, spine gains are especially localized in regions encompassing the border between different barrel columns. These experiments illustrate on one hand how the developing brain is much more plastic than the adult one, and on the other hand, that the type and severity of sensory deprivation critically influence the formation and abandonment of connections.

### Visual Deprivation

Another type of sensory deprivation task is the blocking of visual input. The binocular visual cortex in a given hemisphere receives sensory input predominantly from the contralateral eye, while the contribution of the ipsilateral eye is much lower. This is commonly referred to as ocular dominance (OD). Depriving visual input of one eye, also called monocular deprivation (MD), and recording visual signals in the contralateral visual cortex show increased responsiveness towards the non-deprived, ipsilateral eye while the deprived eye responses fade (Shatz and Stryker, 1978). This is called OD plasticity, which is maximal during a critical period in young mice and requires longer-lasting MD in adults, typically during at least 5 days (Gordon and Stryker, 1996). However, OD plasticity upon short-lasting MD can be reinstated in adult mice under the condition that another, OD plasticity-inducing MD episode took place earlier in the life of the animal (Kind et al., 2002; Hofer et al., 2006). Thus, a transient adaptation to altered visual input leaves a “trace” in cortical circuits that facilitates similar adaptations in the future. To investigate whether and how morphological alterations in dendritic spines could participate in this memory trace, *in vivo* TPM has been used to observe spine dynamics during OD plasticity. The Huebener group studied dendritic spine turnover in adult mice submitted to two subsequent episodes of MD (Hofer et al., 2009). During the first deprivation, binocular visual cortex neurons increased spine formation, while spine elimination remained unchanged, leading to a net spine gain. Interestingly, newly formed spines persisted but shrunk during the recovery phase in between the two deprivation episodes (Hofer et al., 2009). The second deprivation did not modify spine dynamics or density but selectively re-enlarged the spines formed during the first deprivation. Thus, spines added during the first MD may provide a structural basis for subsequent OD shifts. These data point out a strong link between functional plasticity and specific synaptic rearrangements, revealing a mechanism of how prior experiences could be stored in cortical circuits through specific spines. Of note, binocular deprivation in juveniles and adults has not been connected to any significant changes in spine turnover (Majewska and Sur, 2003; Zhou et al., 2017). This indicates that sensory competition between contralateral and ipsilateral inputs is required to modify spine turnover.

### Motor Learning

The idea that selected dendritic spines represent a structural memory trace of specific experiences can be challenged in experiments involving learning and the retention of certain skills. Researchers in the field have been using motor learning tasks, such as the paw reaching task, where the animal learns how to reach a food pellet by passing its paw through a narrow gap (Xu et al., 2009), or the accelerated rotarod learning task, which requires the mouse to find balance on a rotating cylinder (Yang et al., 2009). Mice exposed for the first time to the paw reaching task rapidly formed new spines within the contralateral forepaw motor cortex (Xu et al., 2009). As approximately half of these new spines stabilized over 8 days (which is higher than the average spine survival in basal conditions), the general motor performance of the animal concomitantly increased, and the level of performance correlated with the amount of retained new spines. Prolonging training not only stabilized the newly-formed spines long term but also increased the elimination of preexisting spines compared to baseline elimination rates, resulting in a total spine density that eventually matched control levels (Xu et al., 2009). In another study, Yang et al. (2009) subjected mice to the rotarod task and made similar observations. Rotarod learning led to new spine formation and elimination of pre-existing spines. The survival rate of learning-induced new spines and the elimination rate of pre-existing spines both increased with the training duration and were long-lasting. The extent of spine remodeling correlated with behavioral improvement after learning, suggesting a crucial role of synaptic structural plasticity in memory formation. Trained animals were able to maintain successful motor performance in the long term, even if they did not execute the task for months, indicating that stable learning-induced spines might underlie the controlled execution of specific motor skills (Xu et al., 2009; Yang et al., 2009).

Thus, successful retention of a motor task does not only require the formation of new synaptic contacts but involves equally permanent removal of some pre-existing spines. These time-dependent spine density changes contribute to the creation of a neuronal network that serves as the foundation of durable motor memory. Overall, these studies indicate that learning and daily sensory experience leave small but permanent marks on cortical connections and suggest that lifelong memories can be stored in largely stably connected synaptic networks (Yang et al., 2009; Figure 1).

### Sleep

Maret et al. (2011) studied the effect of sleep on L5 apical tuft spine dynamics in adolescent Thy1-GFP mice using a thinned skull approach. Interestingly, they found that spine elimination over a 24-h circadian cycle preferentially occurs during sleep. More specifically, during sleep spine elimination overcomes spine formation, leading to a net spine loss, while conversely, spine formation exceeds elimination during wakefulness, such that over a complete 24 h cycle net spine number remains stable. Moreover, daily spine elimination is down-tuned in sleep-deprived mice (Maret et al., 2011). On the other hand, it was shown that sleep after motor learning promotes the formation of dendritic spines on a subset of branches specific for this task (Yang et al., 2014). Sleep deprivation directly after a motor training inhibited learning-induced new spine formation and resulted in reduced retention of motor memory. This is in line with the idea that sleep contributes to memory consolidation. Notably, using Ca^2+^ imaging the researchers further described that cells active during the motor task are reactivated during sleep, and disrupting this neuronal reactivation prevents learning and branch-specific spine formation. Another study from the same team further showed that natural elimination of a fraction of newly formed spines in response to a motor task happens in the hours following training, notably during sleep (Li et al., 2017). Deprivation of REM sleep eliminates fewer task-associated new spines compared to control mice. This indicates that REM sleep is necessary for the selective elimination of presumably unnecessary experience-associated spines, which allows to free up space in neuronal circuits. The work by Li et al. (2017) further demonstrates that the newly-formed task-associated spines that are destined to become stable undergo an increase of size during REM sleep, unlike REM-deprived mice. The latter observation is in line with other seminal studies indicating that sleep is essential for synaptic scaling (Diering et al., 2014; De Vivo et al., 2017). In sum, sleep bidirectionally shapes neuronal circuits, by the elimination of unnecessary spines and strengthening of essential task-associated synapses.

### Fear Conditioning and Extinction

Changes in spine turnover can be caused by complex environmental influences, for example, such that trigger fear. One extensively studied paradigm is fear conditioning. Rodents typically respond to a foot shock with freezing behavior. If the foot shock, called unconditioned stimulus (US), is paired with a neutral stimulus such as a sound or an environmental context (conditioned stimulus or CS), the animal rapidly learns to associate the CS with the US and will now freeze whenever the CS is presented alone (Kim and Jung, 2006). Strikingly, a single conditioning session is sufficient to form immediate and long-lasting fear memories (Poulos et al., 2016). Fear conditioning is encoded in the amygdala (Davis, 1997), hippocampus (Phillips and LeDoux, 1992) and prefrontal cortex (PFC; Quinn et al., 2008), of which hippocampus, anterior cingulate cortex, and infralimbic cortex show an increase of spine densities upon fear induction (Vetere et al., 2011; Pignataro et al., 2013). Reversal of fear conditioning is possible by exposing the test animal numerous times to the CS only; this is referred to as fear extinction. Interestingly, after fear extinction, spine density gets restored to pre-fear conditioned levels in the basolateral amygdala (BLA) *in vitro* (Heinrichs et al., 2013).

Lai et al. (2012) used TPM to monitor the frontal association cortex—a part of the dorsal PFC—of juvenile mice during auditory fear conditioning and extinction. They focused on L5 projection neurons using Thy1-YFP mice (line H). TPM analyses showed that fear conditioning induces a long-lasting increase in spine elimination while spine formation is unaffected (Lai et al., 2012; Chu et al., 2019). In contrast, fear extinction triggers the formation of new long-lasting spines that tend to appear near the location of formerly erased ones, thus re-establishing pre-fear levels of global spine density. Strikingly, repeating the fear conditioning protocol on these animals induces the selected elimination of those spines that were reformed after fear extinction (Lai et al., 2012). This shows that fear conditioning and extinction lead to opposing alterations at the level of selected spines and synapses.

In contrast to the frontal association cortex, neurons in the auditory cortex, which is involved in fear memory recall, respond to fear conditioning by increased spinogenesis, and fear extinction favors the elimination of those new spines (Lai et al., 2018). But conceptually similar to the association cortex findings, extinction in the auditory cortex eliminated the very spines formed by fear conditioning and reconditioning induced reformation of new dendritic spines close to the sites of new spine formation induced by previous fear conditioning. Notably, persisting new spines induced by fear conditioning were auditory cue-specific and clustered within branch segments (Lai et al., 2018). Together, results from the two seminal studies from Lai et al. (2012, 2018) show that fear conditioning, extinction, and reconditioning induce cue- and location-specific dendritic spine remodeling in the frontal association and auditory cortical areas. They also indicate that changes of synaptic connections induced by fear conditioning are reversed after fear extinction, which contradicts prior hypotheses that fear extinction corresponds to a new form of learning (Myers and Davis, 2007).

Another study attempted to identify the specific neuronal input partners that might be responsible for forming axonal boutons onto auditory cortex neurons with altered spine remodeling, using tracing techniques and dual-color TPM (Yang et al., 2016). They discovered that a direct connection between the lateral amygdala and L5 pyramidal neurons in the auditory cortex is involved in the aforementioned dendritic spine plasticity after fear conditioning (Yang et al., 2016). In an elegant setup, they simultaneously imaged amygdalar axonal boutons and dendritic spines in auditory L1 *in vivo* and found that fear-induced synaptic contacts are formed by adding new partners to already existing pre- or postsynaptic elements between these two structures. This resulted in a net increase in both spine and axonal bouton formation.

Although the fear-induced changes in spine dynamics vary highly between different brain regions, they have in common that they are rapid and usually long-lasting without the necessity of repeating fear-inducing experiments. This is consistent with the fact that in the natural world the recognition of potentially life-threatening situations is crucial for survival. Almost all of the formerly presented studies report likewise that the extent of fear-associated changes in spine elimination or formation is directly correlated with the animal’s behavior (Lai et al., 2012; Yang et al., 2016; Chu et al., 2019).

### Stress and Corticosterone

Other environmental stimuli that modify spine dynamics are variations in stress levels. A predominant role is given to glucocorticoids. The main glucocorticoid in humans is cortisol and in rodents corticosterone (Joëls et al., 2018). Corticosterone regulates the stress response by binding two receptors: the glucocorticoid (GR, also called NR3C1) and mineralocorticoid receptors (MR or NR3C2; De Kloet et al., 1998). Acute, short-term stress typically ameliorates physical performance and supports the consolidation of memories (Cordero and Sandi, 2007; Liston et al., 2013). In contrast to acute stress, chronic stress lowers performance (see review by Joëls et al., 2006), especially in memory acquisition and consolidation (Conrad et al., 1996; de Quervain et al., 1998; Mizoguchi et al., 2002).

Concomitantly to their behavioral effects, stresses induced by exposure to external stressors or by corticosterone administration have profound effects on the structure of dendrites and spines across numerous brain areas, such as the PFC (Radley et al., 2006; Liu and Aghajanian, 2008), the somatosensory cortex (Jeanneteau et al., 2018), the motor cortex (Liston and Gan, 2011), hippocampus (Patel et al., 2018), amygdala (Patel et al., 2018) and striatum (Dias-Ferreira et al., 2009). This points to a universal role of glucocorticoids in the dendritic and spine morphogenesis in the brain. *In vivo*, TPM has shown that acute administration of corticosterone in mice induces a rapid increase of both spine formation and elimination in the barrel cortex of juveniles and adult mice (Liston and Gan, 2011). In contrast, prolonging the stressful episode by rendering it chronic leads to exaggerated spine elimination that largely exceeds spine formation and thus results in strongly reduced net spine density (Liston and Gan, 2011). Further, chronic stress also induces the elimination of pre-existing stable spines that were not affected by short episodes of stress (Liston and Gan, 2011). Recently, these structural spine changes were shown to causally underlie chronic stress-induced behavior, at least in the PFC (Moda-Sava et al., 2019); this is detailed in the discussion below.

As all these dendritic and spine phenotypes have been observed both by exposing the animal to a stressful situation or by administering corticosterone, it is evident that the stress hormone plays a major role in dendrite and spine remodeling. This is further supported by the fact that blocking GR or MR significantly modulates spine formation and elimination over 24 h (Liston and Gan, 2011). Applying an MR antagonist reduces spine formation and elimination by approximately 75%, while a GR antagonist lowers only spine formation without influencing elimination rates. Liston et al. (2013) used transcriptional inhibitors and were able to identify that spine elimination is most likely modulated *via* MR and transcriptional pathways, whereas spine formation depends on faster non-transcriptional processes. Nonetheless, the exact underlying signaling pathways of stress-induced spine dynamics and stabilization are so far incompletely delineated. They most likely depend on a combination of distinct transcriptional and non-transcriptional actions and the activity of complex co-regulatory elements (Weikum et al., 2017; see discussion below). Notably, besides stress, age-related cognitive decline is similarly associated with dendritic atrophy and spine loss (reviewed by Dickstein et al., 2013), raising the possibility that glucocorticoid signaling might also participate in this process.

### Spatial Confinement of Experience-Induced Spine Changes

We have seen that remodeling of dendritic spines accompanies the learning of motor tasks (Xu et al., 2009), fear-inducing experiences (Yang et al., 2016), and new sensory inputs (Yang et al., 2009). Interestingly, such experience-induced spine remodeling occurs at non-random locations on dendrites as they tend to spatially cluster. First, several studies report clustered spine formation in spatial proximity at sites of synaptic potentiation. De Roo et al. (2008) described that LTP induction by theta-burst stimulation leads to the formation of new functional spines close to activated spines in slice preparations. Fu et al. (2012) showed by *in vivo* TPM of the motor cortex that after repeated training of a specific motor task many of the new task-associated spines form entirely new clusters or clusters near already existing spines. Moreover, they revealed that clustered spines are stable for the long term as opposed to non-clustered spines, even if the associated motor task training stops. A similar *in vivo* experiment by Frank et al. (2018) demonstrated as well that more than 42% of nascent spines appeared in clusters after repeated episodes of contextual learning in the retrosplenial cortex and likewise, these clusters remained largely stable over weeks following the initial learning task.

Consistent with the observed clusterization of spines devoted to the same task, it was also shown that certain motor tasks tend to activate selected branches on the apical dendrite of a given neuron. Indeed, *in vivo* Ca^2+^ imaging showed that dendritic segments of the same neuron generate branch-specific Ca^2+^ spikes with little to no overlap in response to even subtly different motor tasks. Furthermore, spines that happen to be active at the moment of branch-specific Ca^2+^ spikes, undergo functional potentiation (Cichon and Gan, 2015). This indicates that spine formation and synaptic potentiation do not only cluster on a given dendritic branch but are also enriched in specific dendritic branches compared to sister branches (separated by a node on the same neuron). In sum, spatially clustered spines appear to participate in the same task or memory-related circuit. Upon repeated activation of the corresponding circuit, these grouped synapses are potentiated and the associated spines become stable over a long period.

## Dysregulation of Spine Dynamics in Neuropathologies

In this review, we focus on neuropsychiatric disorders. Dendritic spine abnormalities in neurodegenerative disorders have been treated in the review by Herms and Dorostkar (2016). The first indications that mental illnesses were based on abnormal spine numbers and morphology came from the analysis of human post-mortem tissues. Patients diagnosed with intellectual disability (ID) showed reduced spine density and abnormal long and thin spines on apical dendrites of pyramidal neurons in the motor cortex (Purpura, 1974). Reduced spine density was also reported in the auditory cortex of patients with schizophrenia (SCZ; Sweet et al., 2009) and in the PFC of patients with bipolar disorder (BD; Konopaske et al., 2014). On the other hand, autism spectrum disorders (ASD)—a term comprising disorders involving developmental deficits in social interaction, communication and the appearance of repetitive behaviors and stereotypies (Rapin, 1997)—are generally associated with increased spine densities, at least across the temporal lobe (Hutsler and Zhang, 2010). For a complete review of spinopathies in neurodevelopmental disorders, please refer to Forrest et al. (2018).

Although spine morphological abnormalities have been well described in most neurodevelopmental disorders, the molecular pathways that trigger irregular spine turnover are largely unknown. While the genetic component plays an important role, the environmental impact on the onset and severity of mental diseases cannot be neglected as they add tremendously to the final neurofunctional- and behavioral outcome (Chini et al., 2020). Yet, genetic mouse models of disorders allow testing hypotheses about the molecular pathways. In any case, the fact that spine alterations are the main convergence point between neurodevelopmental disorders raises the general hypothesis that spinopathies are an underlying cause.

### ASD-ID

ASDs have been classified into syndromic and nonsyndromic, a distinction that is based on clinical criteria (Sztainberg and Zoghbi, 2016). In “syndromic” ASD the autistic phenotypes occur in conjunction with additional phenotypes and/or dysmorphic features. The etiology in most syndromic ASD cases is known and can involve chromosomal abnormalities, copy number variations, and mutations in a single gene, such as in fragile X syndrome, Angelman syndrome or tuberous sclerosis complex. The term “nonsyndromic” typically refers to “classic autism,” in which no additional symptoms are present. For most nonsyndromic ASD cases the etiology is unknown, and the term “idiopathic autism” has been used alternatively. Interestingly, ASD and ID likely have overlapping origins as 8–20% of ID patients also meet the criteria for ASD (Kaufman et al., 2010) and 50–80% of ASD patients display ID (Simonoff et al., 2008). Despite a tremendous research effort in the field, only a few high confidence genes or copy number variations responsible for ASD have been discovered, most of which are associated with syndromic ASDs. Some of the best-studied syndromic ASD genes are *FMR1* (Fragile X syndrome), *TSC* (Tuberous sclerosis), and *UBE3a* (Angelman syndrome; Bourgeron, 2015; Sztainberg and Zoghbi, 2016). One well studied syndromic ASD deletion is the 22q11 microdeletion (Di George syndrome).

### Fragile X Syndrome: FMR1

Mutations of the activity-dependent RNA-binding protein FMRP, encoded by the *FMR1* gene found on the X chromosome, cause Fragile X syndrome, a disorder associating ID and ASD. *Fmr1* knockout mice show hyperactivity and abnormal social interactions (Bernardet and Crusio, 2006). Consistent with findings in brain tissue from Fragile X syndrome subjects (Irwin et al., 2001), studies on L5 cortical neurons found that *Fmr1* knockout mice display increased spine densities with immature, abnormally elongated, spine morphologies even at adult ages (Comery et al., 1997; Nimchinsky et al., 2001; Galvez and Greenough, 2005). Conversely, other studies reported no change in spine shape or density in L5 as well as other neuronal types (Harlow et al., 2010; Till et al., 2012; Wijetunge et al., 2014). These differences may be attributed to age, the brain area that is examined, the genetic background, and/or methodology. In any case, fixed tissues only provide a snapshot of processes that are in reality dynamic and thus may not capture abnormalities in spine remodeling. In fact, *in vivo* TPM has revealed abnormally high baseline spine turnover ratios in various cortical areas of *Fmr1* knockout mice (Cruz-Martín et al., 2010; Pan et al., 2010; Padmashri et al., 2013; Nagaoka et al., 2016). *In vivo* imaging of L2/3 in the barrel-cortex shows no differences in spine density or morphology between wild type and *Fmr1* knockout mice (Cruz-Martín et al., 2010). The same knockout mice, however, present increased spine turnover and fail to downregulate spine dynamics at 2 weeks of age (Cruz-Martín et al., 2010). This is due to the defective transition from early protrusions to mature spines, entailing that fewer spines undergo stabilization. Pan et al. (2010) observed L5 of the barrel cortex and concluded likewise, that *Fmr1* knockout mice have a larger pool of unstable spines which accounts for the increased dynamics.

Interestingly, while spine turnover is enhanced in *Fmr1* knockout mice in basal conditions, it is much less sensitive to motor learning and experience than in control animals (Cruz-Martín et al., 2010; Pan et al., 2010; Padmashri et al., 2013; Nagaoka et al., 2016). Pan et al. (2010) found that sensory deprivation by chessboard whisker trimming would not induce new spine formation in *Fmr1* knockout mice. Similarly, such mice fail to learn a motor task, as motor training-associated new spines fail to form (Padmashri et al., 2013). Furthermore, *Fmr1* knockout mice do not undergo increased spine formation under an enriched environment as wildtype mice do (Arroyo et al., 2019). Therefore, *Fmr1* deficiency alters experience-dependent spine dynamics and thus behavioral adaptation to the external world.

FMRP malfunction dysregulates the local activity-dependent translation of numerous mRNAs at the synapse (Bassell and Warren, 2008; Sethna et al., 2014). As FMRP is considered a translational repressor, mutations induce an augmentation of synapse-relevant proteins that could act upon spine dynamics (Sidorov et al., 2013). For example, activity-regulated cytoskeleton-associated protein (ARC), a synaptic protein critical for the internalization of α-amino-3-hydroxy-5-methyl-4-isoxazolepropionic acid (AMPA) receptor trafficking at synapses, is one mRNA target of FMRP at the synapses (Waung et al., 2008; Ebert and Greenberg, 2013). For more information regarding the molecular mechanisms of synaptic dysfunction in Fmr1 knockout mice, please refer to Nishiyama (2019).

### Angelman Syndrome: Ube3a

Loss of expression of the ubiquitin-protein ligase E3a (UBE3A) is associated with most cases of Angelman syndrome, which is a rare syndrome of developmental delay, ID and ASD (Buiting et al., 2016). Opposed to loss-of-function mutations, duplications or triplications of the gene are also highly common among patients diagnosed with ASD (Vatsa and Jana, 2018), which points to a highly regulated role of UBE3A in the organism. Greer et al. (2010) identified that UBE3A controls the activity-dependent degradation of ARC in spines, which is involved in the internalization of glutamate AMPA receptors. Consequently, absence or mutation of UBE3A can reduce AMPA receptors at postsynaptic sites and thereby modify excitatory synaptic transmission.

In mouse models of Angelman syndrome that lack *Ube3a*, dendritic spines present abnormal morphologies and reduced densities in the hippocampus and neocortex (Dindot et al., 2008; Yashiro et al., 2009). *In vivo*, TPM allowed gaining more insight into the basal and experience-dependent spine dynamics in such mice. Yashiro et al. (2009) and Kim et al. (2016) described that dendritic spines of *Ube3a* deleted mice undergo excessive pruning while spine formation remains unchanged (Kim et al., 2016), thus resulting in a net loss of spines. However, in *Ube3a* deleted mice that are raised in darkness, spine density and dynamics were indistinguishable with controls, which indicates that decreased spine density in Angelman syndrome model mice reflects impaired experience-driven spine maintenance. The general notion of impaired experience-dependent plasticity in Angelman syndrome is reinforced by the observation that MD, which usually induces an OD shift in the visual cortex of wild type mice, does not have such an effect in *Ube3a*-deleted animals (Yashiro et al., 2009). These abnormalities point to a function of UBE3A in experience-dependent plasticity during development that could play a role in the cognitive deficits observed in Angelman syndrome.

### 22q11 Deletion Syndrome

Another genetic predisposition for SCZ and ASD is chromosomal microdeletions on position 22q11 encompassing up to 40 different genes that can lead to 22q11 deletion syndrome. As numerous genes are affected, patients can present various additional phenotypes as facial dysmorphia, thymic hypoplasia, or cardiovascular anomalies (Squarcione et al., 2013). Nevertheless, there is a strong component for neuropsychiatric disorders: 22q11 deletion syndrome induces SCZ in 30% of patients (Earls and Zakharenko, 2014). As this region is conserved in mice, 22q11 deletion syndrome can be relatively easily modeled to study the physiopathology of the syndrome. Analysis of cultured hippocampal 22q11 deleted mouse neurons shows reduced spine density, smaller mushroom spine heads, and reduced dendritic complexity, suggesting morphological immaturity (Xu et al., 2013). Interestingly, Moutin et al. (2017) observed in hippocampal organotypic cultures that 22q11 deleted neurons present higher spine formation and elimination rates than wild type neurons, such that the total spine turnover is balanced and not responsible for the observed reduced spine density. Instead, they observed decreased long-term spine stabilization. This short-livity eventually drives the observed reduced number of dendritic spines and thus most likely cognitive impairments. The exact genes within the deletion that drive these neuronal changes have not been identified yet. Strong candidates are proteins that are involved in cell metabolism pathways and regulation of neurotransmission, such as COMT, PRODH, or ZDHHC8 (see review by Squarcione et al., 2013), and the micro-RNA mIR-185 (Xu et al., 2013).

### SCZ: The DISC1 Case

SCZ is characterized by psychotic symptoms that include disorganized thoughts, delusions, or hallucinations and, unlike ASD, finds its typical onset in late adolescence. Studies on human tissue describe reduced dendritic spine density (Sweet et al., 2009; Konopaske et al., 2014). Meta-analyses of twin studies allow estimating that the heritability of SCZ is around 81% (Sullivan et al., 2003), indicating a strong genetic component. One important SCZ risk factor is *DISC1* (Mathieson et al., 2011). Originally, a chromosomal translocation of *DISC1* was found in members of a large Scottish family who developed SCZ (St Clair et al., 1990). In neurons, DISC1 acts as a scaffolding protein and associates with a great number of synapse- and microtubule-associated proteins during cortical development and adulthood (Brandon and Sawa, 2011). Hayashi-Takagi et al. (2010) demonstrated that knockdown of DISC1 in cultured rat cortical neurons leads to spine shrinkage. They further determined that DISC1 regulates activation of the Rho-GTPase RAC1 in the PSD, RAC1 being a protein whose activity modulates spine shape through regulation of actin dynamics (see discussion below). The same group went on to determine the signaling pathway downstream of RAC1 that was regulated by DISC1. Chemical inhibition of p21-activated kinases in DISC1-knockdown neurons partially reversed some of the knockdown-induced spine defects (Hayashi-Takagi et al., 2014). Finally, using TPM in the PFC of DISC1 knockdown mice, they confirmed increased spine elimination between P35 and P60 and found that this was reversed by administering a p21-activated kinase inhibitor. These experiments show that DISC1 defects produce a synaptic phenotype reminiscent of the reduced spine density observed in cases of SCZ and that these defects are communicated *via* the RAC1 pathway, which in turn represents a potential target for therapeutic interventions.

### Rett Syndrome: MECP2

Rett syndrome is induced by loss-of-function mutations in the transcriptional regulator gene *MECP2*. MECP2 is an activity-dependent transcriptional repressor protein that acts by binding to methylated CpG dinucleotides and induces remodeling of the chromatin structure (Nan et al., 1997; Amir et al., 1999; Cohen et al., 2011). *MECP2* is an X-linked gene and most affected patients are females, who present stereotypies, motor capability regression and cognitive impairments that reflect in post-mortem brain tissue by reduced dendritic complexity and reduced spine densities in the hippocampus and across all layers of the cortex (Belichenko et al., 1994; Chapleau et al., 2009). Although some of its symptoms at first remind of ASD, Rett syndrome has been classified as a neurodevelopmental disorder, notably due to its critical motor coordination defects. Approximately 95% of Rett syndrome cases are directly linked to *MECP2* mutations, and their phenotypic severity depends on the type of mutation or the pattern of somatic X-chromosome inactivation in the patient (Chahrour and Zoghbi, 2007). Mouse models of Rett syndrome either express point mutations from patients (Cohen et al., 2011) or are *Mecp2* knockouts (Belichenko et al., 2009). Diverse genetic models develop impressively similar phenotypes that resemble human symptoms, including failure to thrive, cognitive deterioration in early postnatal life, and premature death (Chahrour and Zoghbi, 2007). Anatomically, mouse models of Rett syndrome present reduced spine density but also abnormal axonal orientation and dendritic swelling, which also coincides with observations made in humans and thus renders them suitable for studying the disease (Fukuda et al., 2005; Belichenko et al., 2009).

Since Rett syndrome manifests itself in early postnatal life when experience shapes neuronal circuit wiring, and since *Mecp2* is activity-dependently regulated (Cohen et al., 2011), it is hypothesized that *Mecp2* might mediate experience-dependent processes of synapse development. First, loss of *Mecp2* leads to impairments in LTP and LTD, and in a reduced number of glutamatergic synapses and spines in the hippocampus (Asaka et al., 2006; Moretti et al., 2006). Landi et al. (2011) performed TPM over 1 h in the somatosensory cortex of juvenile *Mecp2* knockout mice. They found a reduced number of filopodia, which accounts for reduced protrusion density, and described spine heads as a lot more stable than in wild type mice in terms of volume fluctuations. This is observed during a critical period where spines normally mature and coincides with the disease onset in the mouse model. In adult mice, spine short term motility does not differ anymore between mutant and control animals, as motility naturally declines also in wild type mice. However, the reduced spine density in the mutant persists (Landi et al., 2011).

Overall, the current data show that dysfunctional MECP2 underlies defective spine turnover during a critical window in development, which induces spine loss. The experience-dependency of MECP2’s role in dendritic spine turnover requires further investigations. Molecular mechanisms underlying RTT have been extensively studied in the past decades and are out of the scope of this study (Luikenhuis et al., 2004; Chang et al., 2006; Giacometti et al., 2007; Guy et al., 2007; Larimore et al., 2009).

### Major Depressive Disorder

Major depressive disorder (MDD) is a psychiatric illness that is characterized by low mood and lack of feeling pleasure. MDD patients show altered glucocorticoid levels, which speaks for dysregulation of the HPA axis (Gold et al., 2015). Similarly to what is observed after exposure to chronic stress in mice (see discussion above), individuals with MDD present reduced spine synapses and decreased brain volume, especially in PFC and hippocampus (Hastings et al., 2004; Kang et al., 2012).

To model MDD in animals, chronic stress paradigms are employed, such as social stress (Hollis and Kabbaj, 2014) or chronic mild stress, where the mouse is exposed to phases of unpredictable stressors (Willner et al., 1992). As discussed above, numerous studies including some, with *in vivo* TPM showed that chronic stress in rodents strongly increases spine elimination, notably in the PFC, leading to a reduced spine density (Radley et al., 2006; Liston and Gan, 2011; Moda-Sava et al., 2019). Besides dendritic spine reduction, the MDD rat model of learned helplessness is also associated with reduced PSD-95 protein levels in the hippocampus (Reinés et al., 2008). This is similar to the analysis of human MDD PFC tissue, which also shows reduced protein expression of PSD-95 and synapse-related genes (Feyissa et al., 2009; Kang et al., 2012). The most common antidepressant drugs that are proven to be effective in humans, such as the NMDA-receptor antagonist ketamine (Murrough et al., 2013), also alleviate symptoms in mice as they overturn reduced animal mobility in the tail suspension tests (Cryan et al., 2005). Interestingly, ketamine is also able to restore, at least partially, dendritic spines eliminated by chronic stress in the mouse PFC (Moda-Sava et al., 2019). Also, the other well-known antidepressant fluoxetine similarly restores higher levels of PSD-95 in the hippocampus of stressed rats (Reinés et al., 2008). Strikingly, an elegant experiment took advantage of the paRac1 approach to demonstrate the causal relationship between spine reformation and behavioral recovery induced by ketamine in mice (see discussion below; Moda-Sava et al., 2019).

In sum, spine defects are a convergence point of many neuropsychiatric disorders (Forrest et al., 2018). Further functional analyses of both existing and new models for neuropsychiatric disorders will be essential to uncover generic and specific mechanisms leading to spine pathology. In this quest, state-of-the-art “omics” technologies will be essential to deconstruct the global pathway alterations taking place in model systems. The fact that spine pathology appears before cognitive defects in certain disorders suggests that there are critical periods for treatment to prevent disease onset (Marín, 2016). Furthermore, several mouse models, such *Mecp2* (Luikenhuis et al., 2004; Giacometti et al., 2007; Guy et al., 2007) and *Shank3* (Mei et al., 2016) have also shown that some structural and behavioral deficits can be reversed in adult animals, offering hope for treating human conditions (Ehninger et al., 2008).

## The Causative Role of Spine Dynamics in Learning and Behavior

There is a strong correlation between spine formation/elimination/stabilization and retention of learned tasks (Xu et al., 2009; Yang et al., 2009). However, due to technical limitations, it has long been impossible to assess the causality link between new spines and learning. Hayashi-Takagi et al. (2015) developed a revolutionary approach that finally allowed tackling this question. They developed a photoactivatable form of the Rho GTPase RAC1, known to regulate spine dynamics through the modulation of actin polymerization (Costa et al., 2020), which they called paRAC1. RAC1 normally accumulates in recently formed, nascent spines, and constitutive RAC1 activation leads to spine shrinkage and elimination (Tashiro et al., 2000). The photoactivation form of paRAC1 renders RAC1 constitutively active and thus eliminates RAC1-expressing recently formed spines. With this tool, Hayashi-Takagi et al. (2015) selectively eliminated new spines induced by rotarod learning. They observed that this elimination blocked memory recall, demonstrating for the first time that task-induced spines are causally involved in memorizing the task. Moda-Sava et al. (2019) employed the same technique to demonstrate that ketamine-induced restored spines in chronically stressed mice are causally involved in the maintenance (but not the induction) of behavioral recovery after treatment. They photoactivated virally-expressed paRAC1 in PFC neurons to selectively reverse the positive effects of ketamine on spine formation; by this approach, they found that the newly formed spines are required to sustain ketamine’s antidepressant effects on motivated escape behavior (Moda-Sava et al., 2019). Interestingly, ketamine-induced spine reformation was required for the maintenance of antidepressant effects but not for their initiation, as ketamine’s effects on behavior and cell ensemble activity preceded its effects on spine formation by several hours.

## Molecular Mechanisms of Dendritic Spine Plasticity

Experiments showing that a massive local release of glutamate or GABA can induce the formation of postsynaptic dendritic spines have indicated that presynaptic neurotransmitter release is likely the main trigger for synapse formation, proving that synaptogenesis is an activity-dependent process that likely depends on the presence of presynaptic axonal boutons (Kwon and Sabatini, 2011; Oh et al., 2016). The post-synaptic mechanisms by which synaptic activity modulates the structure of existing spines have been thoroughly investigated, mostly using global or spine-specific long term potentiation (LTP) or long term depression (LTD) paradigms. The capacity of a stimulated spine to display enlargement or shrinkage upon LTP or LTD, respectively, is called structural plasticity (sLTP and sLTD). Although much fewer studies have investigated the molecular control of *de novo* spine formation/elimination, the current evidence indicates that they share similar mechanisms with spine enlargement/shrinkage (Caroni et al., 2012). Spine dynamics are largely controlled by local actin polymerization/depolymerization. Upon stimulation of dendrites and spines, early inducers of initial spine formation or enlargement comprise cascades of activation of actin-binding proteins (ABPs) including CaMKII and Rho GTPases. Later on, local translation at the spine level is induced to maintain the architecture of spines. Even later, activity-dependent transcriptional mechanisms followed by putative synaptic tagging and capture of plasticity-related genes are required for long-lasting stabilization. We briefly review the molecular mechanisms governing these different phases of spine formation. The specific mechanisms underlying spine shrinkage and elimination during LTD are in part redundant with the ones underlying spine formation/enlargement and are the subject of recent reviews (Segal, 2017; Stein and Zito, 2019).

### Actin Underlies Spine Dynamics

The cytoskeleton of the dendritic spine is predominantly composed of actin filaments (Matus, 2000). Actin monomers (globular or G-actin) polymerize to form actin filaments (filamentous or F-actin) *via* the complex interaction with a variety of actin-binding proteins (ABPs). F-actin provides the force necessary for the formation of nascent protrusions and modifications in spine shape, such that actin polymerization/depolymerization is the main determinant of spine structural dynamics. A LTP inducing stimulation increases actin polymerization in spines (Fukazawa et al., 2003; Okamoto et al., 2004; Lin et al., 2005a). Importantly, actin networks are associated with the PSD, which contains hundreds of proteins that organize and stabilize synaptic receptors, cytoskeletal proteins, and signaling proteins (Kasai et al., 2003; Carlisle and Kennedy, 2005; Ethell and Pasquale, 2005). Also, recent genetic studies have shown that many mutations associated with neurodevelopmental disorders involve genes encoding regulators of the spine actin cytoskeleton (Borovac et al., 2018), validating the hypothesis that mechanisms regulating the actin cytoskeleton may contribute to spine pathology in neurodevelopmental disorders. For a more complete review of actin and ABPs in spinogenesis, please refer to Costa et al. (2020) in this issue of Frontiers in Synaptic Neuroscience, and Borovac et al. (2018).

### Initiation of Spine Enlargement

**Figure 2:**
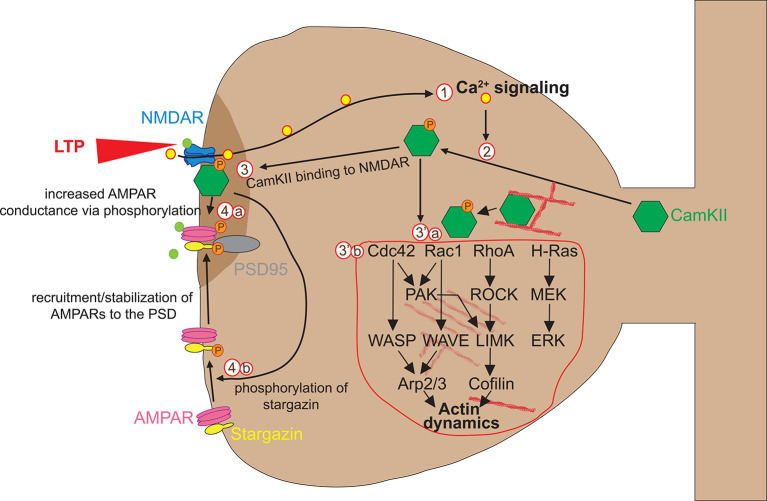
Early phase of spine enlargement/formation. Calcium flowing into the spine through NMDAR (1) binds to the Ca^2+^-binding protein calmodulin (CaM) and Ca^2+^-CaM complex activates CaMKIIα by allowing its auto-phosphorylation (2). Activated CaMKII has two functions in the early stages of spine plasticity: a kinase function (3) and a structural function on actin dynamics (3′). Concerning the kinase function, activated CaMKII translocates to the postsynaptic density (PSD) where it forms complexes with NMDA receptor and other PSD molecules, allowing its stabilization. There, CamKII kinase activity enhances AMPAR-mediated transmission in two ways. First, CaMKII phosphorylates AMPA receptors, which leads to an increase in the average conductance of such channels (4a). Second, CaMKII phosphorylates the AMPAR auxiliary protein stargazin, which causes stargazin to bind PSD95, thereby increasing the number of AMPARs at the synapse (4b). Concerning its structural impact on actin remodeling in spines, CamKII plays a dual function. First, CamKII binds actin directly in its basal state and transiently detaches when phosphorylated to allow F-actin assembly/disassembly that is necessary for actin reorganization underlying spine enlargement (3′a). Second, CamKII activates *via* unknown mechanisms numerous small GTPases including Cdc42, Rac1, RhoA, and H-Ras (3′b). Those small GTPases in turn activate ABPs such as Cofilin and Arp2/3 *via* several kinase pathways, which in turn regulate structural LTP *via* actin remodeling. For more detailed information about the regulation of the spine actin cytoskeleton please refer to Nishiyama and Yasuda (2015).

Glutamate binding to NMDAR triggers rapid Ca^2+^ accumulation, which can be visualized by combining fluorescent Ca^2+^ indicators with live TPM (Higley and Sabatini, 2012). Glutamate uncaging experiments have shown that Ca^2+^ accumulation lasts short (0.1 s) and is mainly restricted to the stimulated spine (Mainen et al., 1999; Sobczyk and Svoboda, 2007), suggesting that dendritic spines can act as independent signaling compartments. Calcium flowing into the spine through NMDA receptors binds to the Ca^2+^-binding protein calmodulin (CaM), and the Ca^2+^-CaM complex then activates the holoenzyme CaMKII that is necessary and sufficient for LTP induction (Lisman et al., 2012). CaMKII is one of the most abundant proteins in neurons and plays a primary role in spine plasticity, learning, and memory. After activation by autophosphorylation, CaMKII rapidly translocates from the parent dendrite to the stimulated spine. Activated CaMKII has two functions in the early stages of spine plasticity: a kinase function on AMPA receptors and a structural function on actin dynamics (Figure 2).

Concerning the kinase function, activated CaMKII translocates to the postsynaptic density (PSD) where it forms complexes with NMDA receptor and other PSD molecules, allowing its stabilization. There, CamKII kinase activity enhances AMPAR-mediated transmission in two ways. First, CaMKII phosphorylates AMPA receptors, which leads to an increase in the average conductance of such channels (Lisman et al., 2012). Second, CaMKII phosphorylates the AMPAR auxiliary protein stargazin, which causes stargazin to bind PSD95, thereby increasing the number of AMPARs at the synapse (Tomita et al., 2005; Opazo et al., 2010; Figure 2).

These processes are confined to stimulated spines and are thought to account for the synapse-specificity of LTP expression, although the causal relationship between CaMKII-derived modulation of AMPA receptor conductivity/synaptic capture and LTP expression remains to be proven.

Concerning its structural impact on actin remodeling in spines, activated CamKII plays a dual function. First, inactive CamKII binds actin directly and transiently detaches when activated to allow F-actin assembly/disassembly events that are necessary for actin reorganization underlying spine enlargement (for more details, see Borovac et al., 2018). Second, CamKII activates mechanisms numerous small GTPases including Cdc42, Rac1, RhoA, and H-Ras to reorganize actin networks in the spine. This was demonstrated thanks to the introduction of FRET and two-photon fluorescence lifetime imaging microscopy (2pFLIM), which made it possible to study dynamic signaling responses in stimulated spines at least in acute slice paradigms (Nishiyama and Yasuda, 2015; Nishiyama, 2019). CaMKIIα activity in individual stimulated spines has been imaged using 2pFLIM of a FRET-based biosensor (Okamoto et al., 2004; Lee et al., 2009). LTP induction by glutamate uncaging triggers rapid activation of CaMKIIα that is restricted to the stimulated spine. CaMKIIα activity decays with a time constant of ~10 s, 100 times longer than the Ca^2+^ transient, suggesting that CaMKII plays a role in prolonging Ca^2+^ initiation signal in the spine. Downstream of CaMKII, Ras, RhoA, Cdc42, and Rac1, are key regulators of actin cytoskeleton dynamics, spine morphogenesis, and LTP (Nishiyama and Yasuda, 2015; Nishiyama, 2019). These signaling proteins cycle between an inactive GDP-bound form and an active GTP-bound form. Guanine nucleotide exchange factors (GEFs) stimulate Rho GTPase enzymatic activity by catalyzing GDP-GTP exchange, whereas GTPase-activating proteins (GAPs) inhibit their activity by catalyzing the hydrolysis of GTP to GDP (Negishi and Katoh, 2005). Using 2pFLIM, the Rho GTPase H-Ras has recently been discovered as a major downstream effector of CaMKII in actin reorganization for structural spine plasticity (Harvey et al., 2008). Indeed, the activity of H-Ras was found rapidly increased at stimulated spines but suppressed after CaMKII inhibition (Harvey et al., 2008). Furthermore, in contrast to CaMKII that stays restricted to the stimulated spine, H-Ras activation spreads along the parent dendritic shaft for over 10 μm. For H-Ras, the spatiotemporal activity of Rac1, RhoA, and Cdc42 has been measured using 2pFLIM of FRET biosensors. These studies show that while like CamKII and Cdc42 activities remain highly restricted to the stimulated spine, Rac1 and RhoA activities, like H-Ras, spread into the dendrite and neighboring spines (Murakoshi et al., 2011; Hedrick et al., 2016). Although hypothetic at this stage, the spread of activated H-Ras or other Ras family members such as Rac1 and RhoA during induction of structural plasticity at the stimulated spine may “predispose” neighboring spines or spine sites for heterosynaptic plasticity (Van Bommel and Mikhaylova, 2016; Hedrick and Yasuda, 2017). It is tempting to speculate that new spine clustering or branch specificity during repetitive task learning might be facilitated by such mechanisms (Lai et al., 2012). One should keep in mind that the aforementioned 2p-FLIM-FRET studies dealt with structural potentiation of existing spines, not with *de novo* spine formation from smooth stretches of the dendrite. Another limitation of these studies is that for technical reasons they were performed on acute slices rather than *in vivo*. A single study has applied the 2p-FLIM-FRET approach *in vivo* in the context of sensory deprivation in the visual cortex (Mower et al., 2011). Although this study provides a proof-of-principle that FRET studies can theoretically be done *in vivo*, Spatio-temporal resolution is lower than in slices, which might in part explain why such *in vivo* experiments have not been reproduced. Finally, it remains to be determined by which mechanisms CamKII activates small GTPases.

Downstream effectors of small GTPases are several kinases including p21-activated kinase (PAK), Rho kinase (ROCK), and LIM kinase (LIMK; Murakoshi et al., 2011). These kinases ultimately activate numerous ABPs including Cofilin and Arp2/3 that play essential roles in actin reorganization. The mechanisms by which ABPs induce actin reorganization upon synaptic potentiation have been abundantly studied in other reviews (Borovac et al., 2018; Costa et al., 2020).

In sum, activation of Rho GTPases and associated ABPs *via* CaMKII activation controls actin polymerization, leading to profound and rapid (within minutes) structural changes at single stimulated dendritic spines. A growing number of genetic studies have linked neurodevelopmental disorders to various synaptic GEFs and GAPs for Rho GTPases (Hamdan et al., 2009; Alber et al., 2017; Stressman et al., 2017). Further studies are required to determine how CaMKII, Rho GTPases, and associated GEFs and GAPs participate in spine formation/elimination under physiological learning conditions *in vivo*.

### Spine Stabilization

The long term stabilization of new spines requires specific mechanisms (Subramanian et al., 2019) and is believed to be the structural correlate of long-lasting LTP (also called late-phase LTP; Baltaci et al., 2019). Long term structural plasticity is mediated by NMDA-receptor-dependent and/or by L-type voltage-sensitive calcium channels (L-VSCCActb Limk1Actb)-dependent calcium influx (Ebert and Greenberg, 2013). In contrast to short term structural spine plasticity, long-lasting plasticity requires protein synthesis, *via* local mRNA translation and gene transcription in the nucleus. The main signaling cascade for local translation at spines requires glutamate binding to metabotropic glutamate receptors (mGluR), which triggers protein-synthesis-dependent forms of spine plasticity by activating extracellular signal-regulated kinase (ERK) or mammalian target of rapamycin (mTOR) pathways (Ebert and Greenberg, 2013). Activity-dependent locally translated mRNAs important for spine plasticity include *Camk2a*, *Actb*, or *Limk1*; for a more complete list please refer to Holt et al. (2019).

The neuronal activity also triggers programs of gene transcription that affect dendritic spine development and plasticity on the longer run (Cohen and Greenberg, 2008; Greer and Greenberg, 2008; Zhai et al., 2013). Activity-dependent gene transcription requires Ca^2+^ signaling (Bading et al., 1993; Dolmetsch et al., 2001; Zhai et al., 2013). Activity-induced calcium entry triggers the activation of several distinct but sometimes converging signaling molecules, including CaMKII, protein kinase A (PKA), MAPK, or the phosphatase calcineurin pathways, each of which phosphorylates or dephosphorylate multiple transcriptional regulators within the nucleus. The best-studied activity-regulated transcriptional regulator is CREB, which upon phosphorylation at Ser 133 by such calcium-dependent pathways activates gene transcription that promotes spine development (Cohen and Greenberg, 2008; Greer and Greenberg, 2008). Other known activity-dependent transcription factors include myocyte enhancer factor 2 (MEF2), serum response factor (SRF), or CREST (Norman et al., 1988; Aizawa et al., 2004; Flavell et al., 2006). Like CREB, other activity-dependent transcription factors such as MEF2 and SRF/ELK are constitutively expressed, and their activation depends on their ability to integrate signals from multiple calcium-dependent pathways *via* post-transcriptional modifications, such as phosphorylation (Cohen and Greenberg, 2008; Greer and Greenberg, 2008; Ebert and Greenberg, 2013). The literature on the signaling mechanisms triggering activity-dependent transcription has been comprehensively reviewed elsewhere (Deisseroth and Tsien, 2002; Lonze and Ginty, 2002; Flavell and Greenberg, 2008; Hagenston and Bading, 2011; Benito and Barco, 2015).

**Figure 3:**
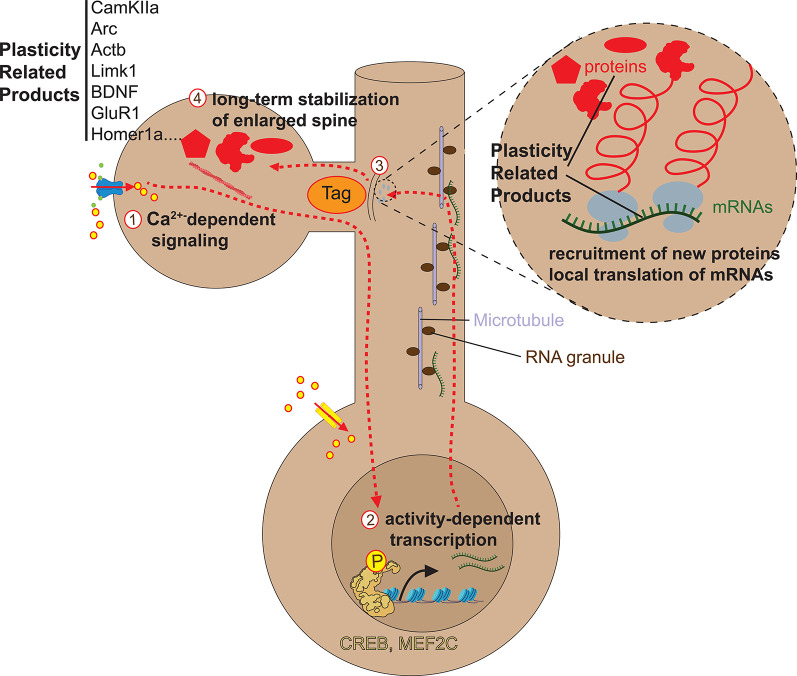
Late phase of spine enlargement/formation. Glutamate release at synapses can induce long-lasting forms of synaptic plasticity that are mediated by NMDA-receptor and/or L-VSCC dependent calcium influx (1) and typically require activity-dependent protein synthesis, which is a consequence of the local mRNA translation within the dendrite (3) or of gene transcription within the nucleus (2,3). The mechanisms underlying activity-dependent local mRNA translation are currently unclear. Concerning gene transcription in the nucleus and transport of new mRNAs/proteins to activated spines, Ca^2+^ influx induces a cascade of kinase/phosphatase signaling pathways that propagate from the spine to the nucleus to phosphorylate/dephosphorylate activity-dependent transcription factors such as CREB or MEF2c. In turn, these factors induce the gene expression of plasticity-related products (PRPs) in the cytoplasm, either proteins or mRNAs. These PRPs are then transported and selectively captured by stimulated spines *via* a synaptic tagging and capture mechanism whose precise nature is still debated (3).

Activity-dependent transcription factors, once phosphorylated by calcium signaling pathways, immediately activate an early transcriptional program corresponding to immediate early genes (IEGs), such as *Fosb*, *Fosl1*, *Fosl2*, *Jun*, *Junb*, *Egr1*, *Egr3*, and *Nr4a1* (Lyons and West, 2011). These IEGs then induce a program of late-response genes (LRGs) that will provide new spines with plasticity-related proteins (Hrvatin et al., 2018; Yap and Greenberg, 2018). One big question is how only stimulated spines can be selectively provided with plasticity-related proteins when activity-induced transcription typically changes gene expression in the whole cell. A body of studies indicates that activity-dependent mRNAs and proteins can preferentially be transported and captured at stimulated spines for local translation *via* a synaptic tagging and capture mechanism (Figure 3) that remains to be elucidated (Martin et al., 1997; Martin and Kosik, 2002; Redondo and Morris, 2011; Okuno et al., 2012; Pinho et al., 2020).

Numerous neuronal activity-dependent LRGs have been characterized (Loebrich and Nedivi, 2009; Leslie and Nedivi, 2011), but only a few genes have been linked with structural spine dynamics. A recent example is *Cpg15* (for Candidate Plasticity Gene 15, also known as *Neurontin*), an activity-regulated gene highly expressed at developmental times of synaptogenesis (Nedivi et al., 1996; Corriveau et al., 1999; Lee and Nedivi, 2002). *Cpg15* KO mice show defects in synapse formation (Fujino et al., 2011). Recently, *in vivo* TPM in the visual cortex showed that CPG15 is not required for rapid spine formation (Subramanian et al., 2019). Surprisingly, visual experience was also not required. However, PSD95 recruitment to nascent spines for their subsequent stabilization requires both visual input and CPG15. Notably, elegant experiments using conditional deletion in *Cpg15* floxed mice showed that CPG15 is necessary post-synaptically for spine stabilization. Further, CPG15 is not only required but sufficient for spine stabilization as its forced expression in post-synaptic neurons compensates for visual deprivation in allowing spine stabilization. Mechanistically, the data indicate that CPG15 physically interacts with AMPA receptors at the nascent spine and then recruits PSD95 for stabilization.

Many of the proteins that constitute the activity-dependent signaling network controlling gene transcription are implicated in neurodevelopmental disorders, in particular ASD (Ebert and Greenberg, 2013; Yap and Greenberg, 2018). This suggests that dysregulation of activity-dependent signaling networks, in general, may contribute significantly to the synaptic dysfunction that occurs in such neurodevelopmental disorders.

### “External” Triggers of Spinogenesis

The current knowledge based on uncaging experiments states that the principal initial triggers for spinogenesis are the binding of the neurotransmitters, glutamate, and GABA through their binding to NMDA and GABA-A receptors, respectively (Kwon and Sabatini, 2011; Oh et al., 2016). Glutamate triggers spinogenesis lifelong, while the spinogenic effect of GABA is restricted to early life when the neurotransmitter is depolarizing.

Although neurotransmitter/receptor interactions are essential to determine where spine formation/elimination occurs on dendrite stretches, other external molecules have been recently shown to coregulate spine dynamics. Studies in the ventral tegmental area (VTA) and hippocampus have enlightened the role of the neuromodulators dopamine and serotonin in spine enlargement and elongation. In slices, dopamine secreted by VTA neurons was shown to promote glutamate-induced spinogenesis in nucleus accumbens medium spiny neurons. Researchers optically stimulated dopaminergic and glutamatergic inputs separately and found that dopamine promoted spine enlargement only during a narrow time window (0.3–2 s) after the glutamatergic inputs. The downstream spine effector mechanisms included calcium entry, cAMP, and PKA activation (Yagishita et al., 2014). These data uncover a molecular basis and spine mechanism for the concept of reinforcement of animal behavior. Concerning serotonin, Bijata et al. (2017) have found in dissociated hippocampal cultures a signaling module involving the 5-HT7 receptor (5-HT7R), matrix metalloproteinase 9 (MMP-9), the hyaluronan receptor CD44, and the small GTPase Cdc42. Stimulation of 5-HT7R results in MMP-9 activation, which, in turn, cleaves CD44. This results in local detachment from the extracellular matrix, which facilitates spine elongation.

The predominant influence of stress and the circadian cycle highlights the critical role of glucocorticoid in spine dynamics. The two receptors for glucocorticoids GR and MR are involved in glucocorticoid actions (Liston and Gan, 2011; Liston et al., 2013). Tritiated labeling first showed that GR and MR exist as transcription factors bound to genomic DNA (Sarrieau et al., 1984; Alexis et al., 1990). However, EM analyses indicated that GR and MR can also be found at the cell membrane, in particular at pre and postsynaptic sites (Prager et al., 2010). The canonical model of action of GR and MR upon glucocorticoid binding is to activate a specific gene transcription program. This program can be triggered either by DNA-bound GR/MR since glucocorticoid can cross cell and nuclear membranes, or by synaptic GR/MR that can translocate to the nucleus after glucocorticoid binding. Strikingly, a non-transcriptional role of GR has been shown in the rapid formation of nascent spines *in vivo*, already 1 h after local corticosterone infusion (Liston et al., 2013). Molecular analyses indicated that activation of dendritic GR initiates spine formation through local interaction with the LIMK1-cofilin pathway and subsequent modulations of actin polymerization. Nevertheless, glucocorticoid-induced new spines then tend to stabilize and survive long term, which requires longer-lasting, transcriptional mechanisms that largely remain to be determined (Leslie and Nedivi, 2011). In contrast to GR, pharmacologic manipulations indicate that MR is predominantly involved in spine elimination and that the mechanisms at play are purely transcriptional (Liston et al., 2013). To add a level of complexity, recent studies have indicated that the transcriptional activities of GR and MR upon glucocorticoid activation require the interaction with the NEUROD family of bHLH transcription factors (Van Weert et al., 2017, 2019). In particular genomic DNA binding sites for MR are all found near NEUROD binding sites on genomic DNA, and both MR and GR depend on NEUROD2 for efficient transactivation of their target genes, as demonstrated on a luciferase assay (Van Weert et al., 2017). *Neurod2* is expressed in pyramidal neurons of the cortex and hippocampus starting from their birth up until animal death. Interestingly, NEUROD2 was identified by the elegant “transactivator trap” genetic screen designed by the Ghosh team as an activity-dependent transcription factor, like CREB. Indeed, NEUROD2 transactivation activity is potentiated by neuronal activity in a calcium-dependent manner (Ince-Dunn et al., 2006). Interestingly, a mouse study has suggested that Neurod2 KO mice are insensitive to stress in the elevated plus-maze and fear conditioning box (Lin et al., 2005b). We recently found that NEUROD2 loss-of-function mutations are causally involved in a neurodevelopment syndrome including ASD and ID in humans (Runge et al., 2020). When analyzing *Neurod2* KO mice, we observed alterations of spine densities in apical tuft dendrites of somatosensory L5 neurons. Spine variations differed in juvenile and adult ages: juvenile mice had fewer spines while adult mice more spines compared to wild type controls. *In vivo*, TPM of apical dendrites helped explain these results as it showed abnormally elevated spine formation rates in juvenile mice, while spine elimination was normal, such that formation took over elimination. Whether NEUROD2’s effect on spine dynamics is entirely dependent on glucocorticoid signaling or whether it can act independently as a mediator of activity-dependent gene transcription for late-phase sLTP remains to be determined. Nevertheless, our bulk (Runge et al., 2020) and ongoing single-cell RNA-seq analyses show that plasticity-related post-synaptic genes are the most enriched set of genes among *Neurod2* KO differentially expressed genes (37/227 genes), which suggests that NEUROD2 is a nexus in a gene network that controls spine turnover in the postnatal cortex.

## Conclusion and Perspectives

We have described the current knowledge about the causes and consequences of dendritic spine plasticity, with a particular focus on recent *in vivo* TPM. Such studies have shown that spine plasticity is caused by several forms of functional plasticity and learning, and that, in return, it is causally involved in the storage of the memory of these experiences on the long-term (Hayashi-Takagi et al., 2015; Moda-Sava et al., 2019). Developmentally, spine plasticity is prominent until adolescence and then drops down in adulthood to very low levels. However, the tremendous number of spines in each brain area allows to compensate for this very low-level adult plasticity and can explain a lifelong causative impact on neural network functions and animal’s behavior (Arenz et al., 2008; Houweling and Brecht, 2008; Yang et al., 2009; Figure 1).

In the future, several approaches might be indicated to accelerate knowledge in the field. in utero electroporation of more than a single fluorochrome will allow capturing not only each spine’s morphology and location but also its subtype identity in real-time *in vivo*. As shown by the work of Nedivi and colleagues (Chen et al., 2012; Villa et al., 2016; Subramanian et al., 2019), the ability to visualize PSD-95, *via* the *in utero* electroporation of a PSD95-mCherry construct, revealed that spines fall into two main subtypes corresponding to different maturation stages. The majority of spines (~80% in adults), correspond to mature excitatory synapses, and these contain PSD-95. Most of these PSD-95 positive spines are stable, but, in the rare cases that they lose PSD-95 and disappear, or are formed *de novo* and gain PSD-95, their dynamics result in a persistent synapse gain or loss and permanent circuit rewiring associated with learning and memory, respectively. The remaining 20% of adult spines are PSD-95 negative. Most of them have immature synaptic currents due to low AMPA receptor contents and thus form unstable synapses. PSD-95 negative spines are highly dynamic and mostly transient, rarely gaining PSD-95 or persisting. Their dynamics likely reflect a sampling strategy for searching potential presynaptic partners in the extracellular space, and the rare spines gaining PSD-95 would be the ones inducing permanent circuit rewiring. In sum, the recent *in vivo* TPM experiments have shown that PSD95 is necessary for spine stabilization but not for spine initiation (Subramanian et al., 2019). Interestingly, adult TPM of *in utero* electroporated plasmids can also allow visualizing the dynamics of molecular determinants of spine plasticity in real-time *in vivo*. As a proof-of-principle, two studies from Huganir and colleagues have shown that *in utero* electroporation of a SEP-tagged GluA1 (an AMPA receptor subunit) plasmid can be used to image experience-dependent AMPA receptor trafficking in real-time *in vivo* (Zhang et al., 2015; Roth et al., 2020). Many molecules other than PSD-95 and AMPA receptors are important for spine plasticity (Sala and Segal, 2014; Schreiner et al., 2017), but their *in vivo* role remains to be assessed, possibly *via* similar approaches.

Importantly, the development of 2pFLIM on organotypic slices has allowed exploring the spatiotemporal dynamics of biochemical signaling in dendritic spines, and a proof-of-principle *in vivo* has been published (Mower et al., 2011). One current limitation of this strategy is that it typically requires the over-expression of FRET-based biosensors (Nishiyama, 2019), which likely disrupts native cell signaling and thus limits the applicability of the method. In this regard, the recent development of CRISP-Cas9 based techniques to fluorescently tag endogenous proteins may open better avenues to image endogenous signal transduction without the effects of overexpressed FRET sensors, and this rather *in vivo* than in slice cultures (Mikuni et al., 2016; Suzuki et al., 2016; Nishiyama et al., 2017).

Besides spines, inhibitory synapses can now be imaged longitudinally by TPM of Gephyrin-fused fluorophores. Nedivi et al. (1996) found that, in cortical pyramidal neuron dendrites, ~30% of inhibitory synapses form on dendritic spines (they called them inhibitory spine synapses) while the rest are shaft synapses. Then, by TPM they discovered that inhibitory spine synapses are much more dynamic than dendritic spines and inhibitory shaft synapses (Chen et al., 2012) and that they are repeatedly assembled and removed at persistent sites (Villa et al., 2016). This could provide flexible, input-specific gating of stable excitatory synapses. Studying further the interplay between inhibitory synapse subtypes and excitatory spines has exciting implications for the understanding of cortical network function in health and neuropsychiatric disorders, which often strongly affect inhibitory neurons (Han et al., 2012; Judson et al., 2016; Ip et al., 2018).

Another prospective advance in the field will be to track presynaptic axons and circuits connecting the dendritic spines whose dynamics are observed. The Gan team has nicely shown that axonal boutons are largely stable in the barrel cortex of adult mice (Qiao et al., 2016). However, for all but one study in the field to date (Yang et al., 2016), the identity of axonal inputs that form connections with learning-induced spines have not been searched. The recent improvements in intersectional genetics and retrograde/anterograde tracers should help to address this issue, which will be essential to deconstruct how specific circuits are modulated by experience and disease.

## Author Contributions

AC and KR wrote the article. KR designed the tables. AC made the figures. All authors contributed to the article and approved the submitted version.

## Conflict of Interest

The authors declare that the research was conducted in the absence of any commercial or financial relationships that could be construed as a potential conflict of interest.
